# Partial Multi-Label Feature Selection via Entropy-Weighted Multi-Scale Neighborhood Granular Label Distribution Learning

**DOI:** 10.3390/e28040422

**Published:** 2026-04-09

**Authors:** Yifan Cao, Mao Li, Cong Wang, Shuyu Fan, Ziqiao Yin, Binghui Guo

**Affiliations:** 1 School of Artificial Intelligence, Beihang University, Beijing 100191, China; cao_yifan@buaa.edu.cn (Y.C.); limao@buaa.edu.cn (M.L.); yinziqiao@buaa.edu.cn (Z.Y.); 2 LMIB and SKLCCSE, Beihang University, Beijing 100191, China; zb2342112@buaa.edu.cn (C.W.); 22377192@buaa.edu.cn (S.F.); 3 Beijing Advanced Innovation Center for Future Blockchain and Privacy Computing, Beijing 100083, China; 4 Hangzhou Internation Innovation Institute of Beihang University, Hangzhou 311115, China

**Keywords:** partial multi-label feature selection, multi-scale neighborhood granular, entropy-based fusion, label distribution learning, sparse regression

## Abstract

Partial multi-label feature selection aims to identify discriminative features from data where each instance is associated with an ambiguous candidate label set. Existing methods are typically built upon single-scale modeling assumptions and may fail to fully exploit the multi-granularity structure underlying instance–label relationships. To address this limitation, we propose a novel framework termed PML-FSMNG, which integrates entropy-weighted multi-scale neighborhood granules with label distribution learning. Specifically, multi-scale neighborhood systems are constructed to estimate label distinguishability at multiple structural scales, and Shannon entropy is employed to adaptively fuse scale-specific label distributions into a robust soft supervisory signal. Based on the learned label distribution, an embedded sparse regression model with $\ell_{2,1}$-norm regularization is developed for discriminative feature selection, together with an entropy-regularized adaptive graph learning mechanism to preserve intrinsic geometric structure. Extensive experiments on benchmark datasets demonstrate that the proposed method consistently outperforms several state-of-the-art approaches, validating the effectiveness of multi-scale modeling and entropy-guided adaptive learning under label ambiguity.

## 1. Introduction

Multi-label data are prevalent across a wide range of real-world applications. For example, in text classification, a document may be assigned multiple categories, including “technical”, “entertainment”, and “economic” [1]; in image annotation, a photo may be associated with both “mountains” and “fields” [2]. However, multi-label data are typically characterized by extremely high-dimensional feature dimensions, where a substantial proportion of features are redundant and even irrelevant to the learning task [3,4,5]. Utilizing the raw high-dimensional feature space often leads to the well-known curse of dimensionality, which degrades model generalization and increases computational complexity [6].

Multi-label feature selection, as an effective dimensionality reduction technique, has therefore attracted considerable attention [7,8,9,10]. By identifying and selecting the most informative and label-relevant features, it aims to enhance the effectiveness and predictive performance of multi-label learning models while reducing computational burden [11,12]. However, in real-world scenarios, annotation errors are inevitable, often resulting in mislabeled samples [13]. This issue gives rise to partial multi-label feature selection (PMFS), where each instance is associated with a candidate label set rather than an exact ground-truth label set. Specifically, PMFS aims to identify the key discriminative features from partially labeled data, under the assumption that at least one label in the candidate set is correct, yet the complete set of relevant labels remains undetermined [14,15].

It is worth noting that the challenge of learning from corrupted candidate set in PMFS is conceptually tied to the broader problem of multi-label learning with missing labels [16,17,18,19]. In both settings, the central bottleneck is that the observed label matrix acts only as an imperfect proxy for the true labels. To mitigate the bias introduced by such incomplete supervision, the missing-label literature has explored several principled theoretical routes. One fundamental logic is based on empirical risk minimization (ERM) [16], which typically leverages global structural constraints—such as low-rank trace-norm regularization—to implicitly recover information across the incomplete label space. Another perspective relies on propensity modeling [17], which explicitly estimates the marginal probability of each label being observed and reweights the available samples to correct the observation bias. Furthermore, recent advances have focused on unbiased loss design [18,19], which systematically constructs tailored convex surrogate loss functions to mathematically guarantee that minimizing the empirical risk on corrupted data aligns with minimizing the true risk on fully labeled data.

While these methods mainly focus on correcting supervision bias at the prediction level, PMFS introduces an additional layer of complexity: algorithms must simultaneously disambiguate the noisy candidate sets and identify the most discriminative feature subsets. To tackle this dual challenge, feature selection approaches for partially labeled data can generally be categorized into two paradigms [20,21]. The first treats all candidate labels as ground-truth labels, thereby simplifying model construction while ignoring potential label noise. The second explicitly models label noise within the candidate set and reduces the impact of unreliable labels during feature selection.

Several representative studies follow the first paradigm. For example, Lee et al. developed a filter-based method (SCLS) [22] based on a relevance–redundancy criterion, in which a new redundancy formulation was introduced to alleviate the dominance of accumulated label-wise relevance and avoid repetitive dependency computations. However, SCLS relies on a simplified first-order approximation, failing to capture complex higher-order label correlations. Li et al. proposed an embedding-based feature selection framework (RFSFS) [23] with a robust flexible sparse regularization term, which addresses the limitations of $\ell_{1}$-norm and $\ell_{2,1}$-norm by jointly capturing label-specific sparsity and reducing feature redundancy. Nevertheless, RFSFS relies on a fixed label correlation graph based on initial cosine similarity, which cannot be adaptively updated during learning. Meanwhile, Fan et al. introduced LCIFS [24], which combines manifold learning with adaptive spectral graph construction and redundancy analysis to jointly model label correlations and suppress redundant features. A notable limitation of LCIFS is its high $O(n^{3})$ computational complexity due to expensive matrix inversions, restricting its large-scale applicability. Ultimately, despite their respective methodological designs, a critical common flaw shared by all three methods is that they implicitly assume the correctness of all candidate labels. Because they lack mechanisms to explicitly handle ambiguous candidate labels, they are highly prone to performance degradation in the presence of noisy or unreliable labels.

In contrast, a growing body of work follows the second paradigm. Wang et al. proposed PMLFS [25], which distinguishes reliable from noisy labels via sparsity and label correlation modeling, and integrates manifold regularization with $\ell_{2,1}$-norm for joint label disambiguation and feature selection. However, its reliance on a static, pre-computed similarity graph in feature space severely limits its adaptability. Subsequent studies further exploited low-dimensional modeling to address label ambiguity. In this line of research, several representative approaches have been proposed. For example, Hao et al. developed PML-FSSO [26] by jointly optimizing label and feature subspaces through low-dimensional decomposition and shared coefficient learning. However, forcing a shared coefficient matrix oversimplifies the distinct intrinsic distributions of feature and label spaces. Qian et al. introduced PFNL [13], a manifold-based embedding approach that projects features into a low-dimensional space and iteratively refines label distributions. Yet, prematurely projecting features into a low-dimensional space risks discarding subtle but highly discriminative original information. Sun et al. proposed PMFS-LRS [27], which decomposes the candidate label matrix into low-rank and sparse components with manifold regularization to enhance robustness. Nevertheless, its strict global low-rank constraint fails to capture complex, localized label dependencies. Building upon this framework, Wu et al. proposed PMSNE [28], a unified model that simultaneously handles partial label ambiguity and feature noise via robust label enhancement and sparse reconstruction; however, its label correlation matrix is prone to noise-induced bias, affecting denoising performance. In contrast to low-rank-based methods, Gao et al. proposed a mutual-information-driven label reconstruction strategy, termed PML-FSMIR [29], which enhances feature selection reliability without explicitly imposing low-rank constraints. Despite its effectiveness, this method generally operate under a single-scale modeling assumption, overlooking the potential multi-granularity structure within label relationships.

To address the above challenges, we propose a novel partial multi-label feature selection framework via entropy-weighted multi-scale neighborhood granular label distribution learning, termed PML-FSMNG. The proposed framework leverages multi-scale neighborhood granules to model instance-level semantic structures across multiple structural scales. Specifically, we construct multi-scale neighborhood systems induced by a monotonically increasing sequence of distance thresholds and estimate label distinguishability at each scale. By incorporating Shannon entropy to measure neighborhood label consistency, scale-specific label distributions are adaptively weighted and fused, resulting in a robust soft label representation that captures multi-granular structural information.

To further enhance feature selection performance, we embed the learned multi-scale label distribution into a sparse regression framework with $\ell_{2,1}$-norm regularization, which promotes joint sparsity across labels and improves robustness against noise. Moreover, to preserve the intrinsic geometric structure of the data, we introduce an adaptive graph learning strategy. Instead of relying on predefined similarity graphs, the similarity matrix is dynamically optimized via entropy regularization, enabling flexible and data-driven modeling of instance correlations. The learned graph Laplacian ensures that neighboring instances in the feature space maintain consistent predicted label representations. The main contributions of this work are summarized as follows:

1. We propose a multi-scale neighborhood granule mechanism to estimate label distribution across increasing neighborhood scales, enabling refined modeling of partial label uncertainty.
2. An entropy-driven fusion strategy is designed to adaptively integrate scale-specific label distributions, yielding a robust soft supervisory signal.
3. Building upon the learned label distribution, we construct an embedded sparse regression framework with $\ell_{2,1}$-norm regularization to select discriminative features while enhancing robustness.
4. An entropy-regularized adaptive graph learning scheme is introduced to capture instance correlations and preserve geometric similarity during optimization.

## 2. Preliminary

### 2.1. Problem Formulation

Let $X={\left[x_{1},x_{2},\ldots ,x_{n}\right]}^{⊤}\in R^{n\times d}$ denote the feature matrix with *n* instances and *d* features, and let $Y={\left[y_{1},y_{2},\ldots ,y_{n}\right]}^{⊤}\in {\left\{0,1\right\}}^{n\times q}$ represent the candidate label matrix, where $y_{ij}=1$ indicates that label *j* is a candidate label for instance *i*. The ground-truth label set of each instance is a subset of its candidate labels, which is unknown during feature selection. The goal of Partial Multi-Label Feature Selection is to select a feature subset F⊆{1,2,…,d} of a specified size *k* that maximizes the relevance to the ground-truth labels, while leveraging the available candidate label information *Y*. Since the true labels are unobserved, it is necessary to characterize the uncertainty and relative importance of candidate labels. Instead of treating candidate labels in a binary manner, a more informative representation can be achieved by assigning a real-valued confidence to each label, which naturally leads to the concept of label distribution learning [30].

**Definition 1** 
(Label Distribution Learning)**.** *Label Distribution Learning (LDL) considers a training set*

(1) $$\hat{S}=\left\{\left(X_{i\cdot },D_{i\cdot }\right)|i=1,2,\ldots ,n\right\},$$

*where $X_{i\cdot }$ denotes the feature vector of the i-th instance and $D_{i\cdot }=\left(D_{i1},D_{i2},\ldots ,D_{iq}\right)$ is the corresponding label distribution. Each component $D_{ij}$ reflects the descriptive degree of label j with respect to instance i, subject to the constraints*

(2) $$D_{ij}\geq 0,\;\sum_{j=1}^{q}D_{ij}=1.$$

*The objective of LDL is to learn a model that predicts a real-valued label distribution for each instance, thereby encoding richer supervisory information than binary label assignments.*

### 2.2. Information Theory

Information theory provides fundamental tools for measuring uncertainty and information content, and has been widely used to evaluate the relevance and discriminative capability of features. In particular, Shannon entropy [31] is employed to quantify the uncertainty of a random variable. Moreover, local semantic consistency among instances can be described by a neighborhood-based representation. Accordingly, we provide the formal definitions of Shannon entropy and neighborhood granule [32] as follows.

**Definition 2** 
(Shannon Entropy)**.** *Let X be a discrete random variable taking values in a finite set $X=\{x_{1},\ldots ,x_{n}\}$ with probability mass function $p_{i}=P\left(X=x_{i}\right)$. The Shannon entropy of X is defined as*

(3) $$H\left(X\right)=-\sum_{i=1}^{n}p_{i}\log p_{i},$$

*where 0log0:=0 and the base of the logarithm determines the unit of entropy.*

**Definition 3** 
(Neighborhood Granule)**.** *Given a feature subset F and an instance $x_{i}\in X$, the neighborhood granule of $x_{i}$ with respect to F is defined as*

(4) $$N_{F}^{\theta }\left(x_{i}\right)=\left\{x_{h}|\Delta_{F}\left(x_{i},x_{h}\right)\leq \theta ,\;x_{h}\in X\right\},$$

*where $\Delta_{F}\left(\cdot ,\cdot \right)$ denotes a distance measure induced by the feature set F, and θ is a predefined neighborhood threshold.*

In this work, $\Delta_{F}\left(x_{i},x_{j}\right)$ is computed using the Euclidean distance in the feature space *F*. The neighborhood threshold θ is adaptively determined as the standard deviation of pairwise distances over all instances under the feature set *F*, which enables the neighborhood structure to adjust to data distribution. Intuitively, instances falling into the same neighborhood granule are highly similar to each other and thus difficult to distinguish based solely on distance information. Such local granules provide a natural way to capture instance-level structure and serve as a foundation for subsequent label-related analysis.

## 3. Method

**Figure 1:**
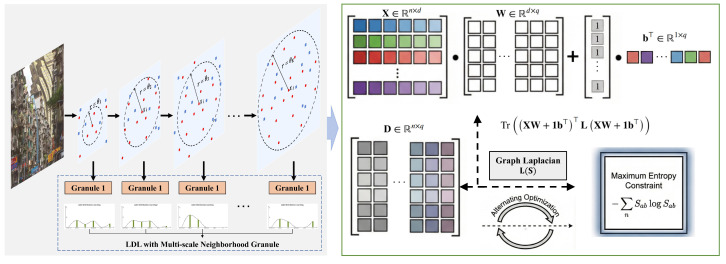
The overall framework of the proposed method PML-FSMNG.

In this section, we introduce the proposed PML-FSMNG framework, which constructs entropy-weighted multi-scale neighborhood granules to model label distributions for partial multi-label feature selection. The overall architecture of the framework is depicted in Figure 1.

### 3.1. LDL with Multi-Scale Neighborhood Granule

Most existing partial multi-label feature selection methods are modeled at a single scale. However, in practical scenarios, the requirements for information understanding are inherently diverse, leading to variations in both the perspective and depth of information processing [33,34]. As a result, analyzing the same object from multiple scales has become a widespread and essential demand. Particularly for Partial Multi-Label data, examining the mapping relationships between features and labels at different scales can yield distinct modeling outcomes and provide deeper insights into the underlying data structure, thereby enhancing the adaptability and generalization capability of models when dealing with partial multi-label data [35]. Based on the above observations, we propose a novel concept termed the multi-scale neighborhood granules, which is defined as follows.

**Definition 4** 
(Multi-scale Neighborhood Granules)**.** *Given a feature subset F and an instance $x_{i}\in U$, let $\Theta =\{\theta_{1},\theta_{2},\ldots ,\theta_{K}\}$ be a set of neighborhood thresholds satisfying $0<\theta_{1}<\theta_{2}<\ldots <\theta_{K}$. The multi-scale neighborhood granules of $x_{i}$ with respect to F are defined as*

(5) $$N_{F}\left(x_{i}\right)=\left\{N_{F}^{\theta_{1}}\left(x_{i}\right),N_{F}^{\theta_{2}}\left(x_{i}\right),\ldots ,N_{F}^{\theta_{K}}\left(x_{i}\right)\right\},$$

*where*

(6) $$N_{F}^{\theta_{k}}\left(x_{i}\right)=\left\{x_{h}|\Delta_{F}\left(x_{i},x_{h}\right)\leq \theta_{k},\;x_{h}\in U\right\},\;k=1,2,\ldots ,K.$$

*Here, $\Delta_{F}\left(\cdot ,\cdot \right)$ denotes a distance measure induced by the feature set F, and each threshold $\theta_{k}$ corresponds to a distinct neighborhood scale.*

**Figure 2:**
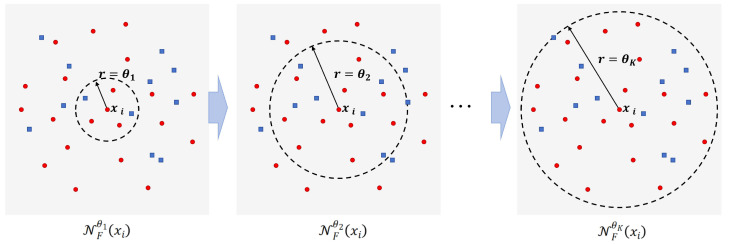
Visualization of multi-scale neighborhood structures under different neighborhood thresholds.

For better intuition, an illustration of the multi-scale neighborhood granules is provided in Figure 2. To capture neighborhood structures from multi-scale perspectives, we adopt a three-scale construction $\left\{\frac{1}{2}\theta ,\theta ,2\theta \right\}$ as a simple yet effective strategy. Specifically, the smallest scale $\frac{1}{2}\theta$ emphasizes fine-grained local variations, the medium scale θ reflects the baseline neighborhood structure, and the largest scale 2θ encodes broader contextual relationships. Such a design is consistent with existing multi-scale analysis methods, where a small number (typically three) of representative scales is commonly adopted to balance modeling capability and computational cost [34,36]. Moreover, although the scale coefficients are fixed, the base parameter θ is adaptively determined according to the data distribution, enabling the resulting multi-scale neighborhood system to flexibly adjust to different datasets and alleviating potential concerns about limited generality.

Based on the multi-scale neighborhood granule system, we first measure the label importance of instances at each scale, and then fuse the label importance obtained from different scales to derive the final label importance. At the *k*-th scale, for any instance $x_{i}\in X$ and label $l_{j}\in Y$, then the positive neighborhood $\delta_{l_{j}^{+},F}^{\theta_{k}}$ of $x_{i}$ as well as the negative neighborhood $\delta_{l_{j}^{-},F}^{\theta_{k}}$ of $x_{i}$ with respect to *F* are as follows:

(7) $$\begin{array}{cc} \delta_{l_{j}^{+},F}^{\theta_{k}}\left(x_{i}\right) & =\left\{x_{h}|Y_{hj}=1,x_{h}\in N_{F}^{\theta_{k}}\left(x_{i}\right)\right\},\\ \delta_{l_{j}^{-},F}^{\theta_{k}}\left(x_{i}\right) & =\left\{x_{h}|Y_{hj}=0,x_{h}\in N_{F}^{\theta_{k}}\left(x_{i}\right)\right\}. \end{array}$$

The distinguishability degree of label $l_{j}$ to instance $x_{i}$ is defined as

(8) $$D_{i,j}^{\theta_{k}}=\left\{\begin{array}{cc} \frac{1}{2}+\frac{|\delta_{l_{j}^{+},F}^{\theta_{k}}\left|-\right|\delta_{l_{j}^{-},F}^{\theta_{k}}|}{2|N_{F}^{\theta_{k}}\left(x_{i}\right)|}, & \mathrm{if}\;y_{ij}=1,\\ 0, & \mathrm{otherwise}. \end{array}\right.$$

Here, in the partial multi-label setting, entries with $y_{ij}=1$ may be noisy, whereas $y_{ij}=0$ is known to be accurate. Therefore, we directly retain $y_{ij}=0$ in the computation of $D_{i,j}^{\theta_{k}}$, ensuring that the label distribution reflects the verified absence of the label while leveraging neighborhood information only for potentially noisy positive labels.

For each sample $x_{i}$ and label $l_{j}$ at the *k*-th scale, we compute the Shannon entropy based on the neighborhood granule $N_{F}^{\theta_{k}}\left(x_{i}\right)$:

(9) $$H_{i,j}^{\theta_{k}}=-p_{i,j}^{\theta_{k}}\log\left(p_{i,j}^{\theta_{k}}\right)-\left(1-p_{i,j}^{\theta_{k}}\right)\log\left(1-p_{i,j}^{\theta_{k}}\right),$$

where $p_{i,j}^{\theta_{k}}=\frac{|\delta_{l_{j}^{+},F}^{\theta_{k}}|}{|N_{F}^{\theta_{k}}\left(x_{i}\right)|}$.

The average neighborhood entropy at scale $\theta_{k}$ is computed by averaging over all samples and labels:

(10) $$H^{\theta_{k}}=\frac{1}{nq}\sum_{i=1}^{n}\sum_{j=1}^{q}H_{i,j}^{\theta_{k}}.$$

Intuitively, smaller $H^{\theta_{k}}$ indicates that labels within the neighborhood are more consistent, reflecting a higher degree of local structural reliability. Conversely, a higher $H^{\theta_{k}}$ corresponds to a more heterogeneous label distribution, implying greater uncertainty. Based on this observation, we define the scale weight $w^{\theta_{k}}$ for each scale $\theta_{k}$ to reflect the reliability of its local label information. Scales with lower neighborhood entropy, indicating stronger feature–label consistency, are assigned higher weights in the multi-scale fusion process, while scales with higher entropy are down-weighted. These weights are normalized across scales using a softmax function over the negative entropy values:

(11) $$w^{\theta_{k}}=\frac{\exp(-H^{\theta_{k}})}{\sum_{k=1}^{K}\exp\left(-H^{\theta_{k}}\right)},\;k=1,2,\ldots ,K,$$

ensuring that $\sum_{k=1}^{K}w^{\theta_{k}}=1$.

**Figure 3:**
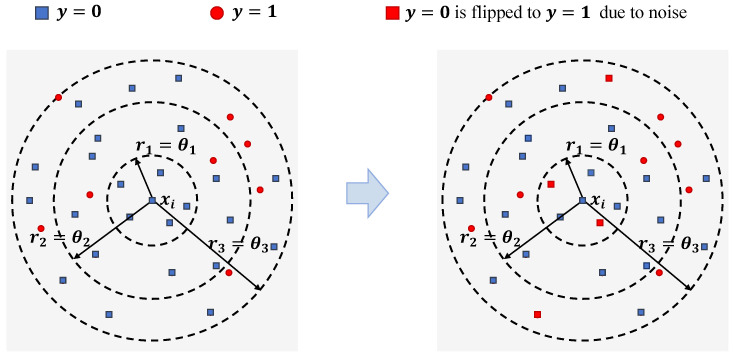
Illustration of multi-scale neighborhoods around an instance $x_{i}$ under noise-free (**left**) and noise-injected (**right**) conditions. In the noise-injected scenario, some instances are flipped from y=0 to y=1, altering the neighborhood label distribution and significantly increasing the local entropy, especially at the smallest scale $\theta_{1}$.

To further illustrate the dynamic adaptation of these weights under varying levels of label noise, we present an example in Figure 3. In this figure, the left and right panels show the local neighborhoods at three scales ($\theta_{1},\theta_{2},\theta_{3}$) under noise-free and noise-injected conditions, respectively. As shown in Figure 3 and quantified in Table 1, under noise-free conditions, the local neighborhood at the smallest scale $\theta_{1}$ is typically more consistent, resulting in zero or low entropy ($H^{\theta_{1}}=0$) and consequently receiving the highest reliability weight ($w^{\theta_{1}}=0.490$). However, when noise is injected, the label distribution at this fine-grained scale becomes highly mixed, causing a sharp spike in entropy ($H^{\theta_{1}}$ rises to 0.918). In response, the weighting mechanism effectively penalizes this uncertainty by reducing $w^{\theta_{1}}$ to 0.321 and adaptively shifting the model’s focus to the relatively cleaner intermediate scale $\theta_{2}$, which then receives the dominant weight ($w^{\theta_{2}}=0.366$).

This illustrative example confirms that this multi-scale weighting mechanism does not rely on the absolute reliability of any single scale. Instead, it integrates structural information from multiple scales: if a particular scale is adversely affected by noise, its neighborhood entropy increases, naturally lowering its weight and reducing its influence in the final aggregation. Finally, once the scale weights $w^{\theta_{k}}$ and the label distributions at each scale $D^{\theta_{k}}$ are obtained, the multi-scale label distribution for each sample is computed as a weighted average across all scales:

(12) $$D_{i,j}=\sum_{k=1}^{K}w^{\theta_{k}}D_{i,j}^{\theta_{k}},$$

where $D_{i,j}^{\theta_{k}}\in R^{n\times q}$ denotes the label distribution matrix at scale $\theta_{k}$, and $w^{\theta_{k}}$ is the corresponding entropy-driven weight. After obtaining $D=[D_{i,j}]$, we further perform row-wise normalization to ensure a valid label distribution:

(13) $$D_{i,j}=\frac{D_{i,j}}{\sum_{j=1}^{q}D_{i,j}},\;j=1,2,\ldots ,q.$$

Thus, for each instance $x_{i}$, it holds that

(14) $$D_{ij}\geq 0,\;\sum_{j=1}^{q}D_{ij}=1.$$

This fusion strategy effectively emphasizes scales with more consistent neighborhood labels, producing a robust soft label matrix that integrates multi-scale neighborhood information. The detailed procedure for constructing the multi-scale label distribution matrix is summarized in Algorithm 1.
**Algorithm 1** LDL with Multi-scale Neighborhood Granule  1:**Input:** Feature matrix *X*, label matrix *Y*, feature subset *F*, neighborhood thresholds $\Theta =\{\theta_{1},\ldots ,\theta_{K}\}$  2:**Output:** Multi-scale label distribution matrix $D\in R^{n\times q}$  3:**for** each scale $\theta_{k}\in \Theta$ **do**  4:      **for** each instance $x_{i}\in X$ **do**  5:            Compute neighborhood granule $N_{F}^{\theta_{k}}\left(x_{i}\right)=\left\{x_{h}|\Delta_{F}\left(x_{i},x_{h}\right)\leq \theta_{k}\right\}$  6:            **for** each label $l_{j}\in Y$ **do**  7:                  Compute label distinguishability $D_{i,j}^{\theta_{k}}$ by (8)  8:                  Compute Shannon entropy $H_{i,j}^{\theta_{k}}$ by (9)  9:            **end for**10:      **end for**11:      Compute average neighborhood entropy: $H^{\theta_{k}}=\frac{1}{nq}\sum_{i=1}^{n}\sum_{j=1}^{q}H_{i,j}^{\theta_{k}}$12:**end for**13:Compute scale weight: $w^{\theta_{k}}=\frac{\exp(-H^{\theta_{k}})}{\sum_{r=1}^{K}\exp\left(-H^{\theta_{r}}\right)}$14:**for** each instance $x_{i}$ and label $l_{j}$ **do**15:      Fuse multi-scale label distribution: $D_{i,j}=\sum_{k=1}^{K}w^{\theta_{k}}D_{i,j}^{\theta_{k}}$16:**end for**17:row-wise normalization: $D_{i,j}=\frac{D_{i,j}}{\sum_{j=1}^{q}D_{i,j}},\;j=1,2,\ldots ,q$18:**Return:** Multi-scale label distribution matrix *D*

### 3.2. Sparse Feature Selection

#### 3.2.1. Basic Model

At this stage, a sparse regression framework is adopted for weight learning due to its strong interpretability and favorable optimization properties. The resulting objective function is formulated as

$$\min_{W,b}{∥XW+1b^{⊤}-D∥}_{2,1}+\alpha {∥W∥}_{2,1},$$

where $W\in R^{d\times q}$ denotes the feature weight matrix and $b^{⊤}\in R^{1\times q}$ is the bias. The $\ell_{2,1}$-norm in the loss term is introduced to alleviate the influence of outliers and enhance robustness, while the $\ell_{2,1}$-norm regularization promotes rowwise sparsity, encouraging the selection of a compact subset of discriminative features shared across multiple labels. α is a regularization parameter that balances the data fitting term and the sparsity-inducing regularization.

#### 3.2.2. Instance Correlation Mining via Graph Representation

When leveraging the predicted label representation $XW+1b^{⊤}$ during the model learning process, it is crucial to preserve the consistency of the geometric structure between the input space X and the prediction space $XW+1b^{⊤}$. Specifically, if two instances $x_{i}$ and $x_{j}$ are close to each other in the input space X, their corresponding predicted label vectors ${\left(XW+1b^{⊤}\right)}_{i}$ and ${\left(XW+1b^{⊤}\right)}_{j}$ should also remain close in the prediction space [37]. To this end, we introduce the following graph-based regularization term to capture instance correlations:

(15) $$\begin{array}{cc} \min_{W,\;b}\frac{1}{2}\sum_{i=1}^{n} & \sum_{j=1}^{n}S_{ij}{∥{\left(XW+1b^{⊤}\right)}_{i}-{\left(XW+1b^{⊤}\right)}_{j}∥}_{2}^{2}\\ \; & =\mathrm{Tr}\;\left({\left(XW+1b^{⊤}\right)}^{⊤}\left(A-S\right)\left(XW+1b^{⊤}\right)\right)\\ \; & =\mathrm{Tr}\;\left({\left(XW+1b^{⊤}\right)}^{⊤}L\left(XW+1b^{⊤}\right)\right), \end{array}$$

where L=A−S denotes the graph Laplacian matrix, A is a diagonal degree matrix with diagonal elements $A_{ii}=\sum_{j=1}^{n}S_{ij}$, and $S_{ij}$ measures the pairwise similarity between instances $x_{i}$ and $x_{j}$ in the input space X.

In many existing methods, instance similarities are captured by predefined graph constructions, such as *r*-nearest neighbor graphs with heat kernel weighting. Although such graph construction strategies are effective in capturing local geometric relationships among samples, the resulting similarity structure is fixed once the neighborhood size and kernel parameter are specified. Consequently, the induced affinity matrix often exhibits a rigid spectral graph structure, which may fail to adapt to the intrinsic data distribution and potentially leads to overfitting during the learning process. To overcome this limitation, we aim to endow the similarity matrix with adaptive capability during feature selection. Inspired by the principle of maximum entropy, we introduce an information-theoretic regularization to dynamically adjust the similarity matrix S in the optimization procedure. Owing to its simplicity and generality, Shannon entropy is adopted to quantify the uncertainty of similarity assignments, and the adaptive similarity matrix S can be obtained by maximizing its entropy:

(16) $$\begin{array}{cc} \max_{S} & \sum_{i=1}^{n}\sum_{j=1}^{n}\left(-S_{ij}\log S_{ij}\right)\;s.t.\sum_{j=1}^{n}S_{ij}=1,\;S_{ij}\geq 0. \end{array}$$

By incorporating entropy regularization, the learned similarity matrix can automatically reflect a reasonable neighborhood structure purely driven by data, without relying on manually specified graph parameters. Accordingly, the adaptive graph modeling of label correlations is formulated as the following joint optimization problem:

(17) $$\begin{array}{cc} \min_{S} & \mathrm{Tr}\;\left({\left(XW+1b^{⊤}\right)}^{⊤}L\left(XW+1b^{⊤}\right)\right)+\gamma \sum_{i=1}^{n}\sum_{j=1}^{n}\left(S_{ij}\log S_{ij}\right)\\ \; & s.t.\sum_{j=1}^{n}S_{ij}=1,\;S_{ij}\geq 0. \end{array}$$

where L denotes the graph Laplacian induced by S, and γ is a trade-off parameter controlling the influence of entropy regularization.

#### 3.2.3. The Objective Function

In summary, the optimization function of the proposed method can be written as:

(18) $$\begin{array}{cc} \; & \min_{W,b,S}{∥XW+1b^{⊤}-D∥}_{2,1}+\alpha {∥W∥}_{2,1}\\ \; & +\beta (\mathrm{Tr}{\left(XW+1b^{⊤}\right)}^{⊤}L\left(XW+1b^{⊤}\right)+\gamma \sum_{i=1}^{n}\sum_{j=1}^{n}\left(S_{ij}\log S_{ij}\right))\\ \; & s.t.\sum_{j=1}^{n}S_{ij}=1,\;S_{ij}\geq 0. \end{array}$$

where α, β, and γ are nonnegative trade-off parameters controlling the relative contributions of different components.

### 3.3. Optimization Solution

#### 3.3.1. Update **b**

By discarding the terms in (18) that are irrelevant to b, the objective function can be written as:

(19) $$\begin{array}{cc} \min_{b}L_{1}= & \;{∥XW+1b^{⊤}-D∥}_{2,1}++\beta \mathrm{tr}\;\left({\left(XW+1b^{⊤}\right)}^{⊤}L\left(XW+1b^{⊤}\right)\right). \end{array}$$

We construct the surrogate function for $L_{1}$:

(20) $$\begin{array}{cc} Q_{1}\left(b|b^{\left(t\right)}\right)= & \mathrm{tr}\;\left({\left(XW+1b^{⊤}-D\right)}^{⊤}G_{0}^{\left(t\right)}\left(XW+1b^{⊤}-D\right)\right)\\ \; & +\beta \mathrm{tr}\;\left({\left(XW+1b^{⊤}\right)}^{⊤}L\left(XW+1b^{⊤}\right)\right), \end{array}$$

where

(21) $$G_{0}^{\left(t\right)}=\frac{1}{2}\mathrm{diag}\left(∥{\left(XW+1b^{⊤}-D\right)}_{i}{∥}_{2}^{-1}\right).$$

By computing the derivative of $Q_{1}$ with respect to b, we obtain:

(22) $$\begin{array}{cc} \frac{\partial Q_{1}}{\partial b}= & b\left(1^{⊤}G_{0}^{\left(t\right)}1+\beta 1^{⊤}L1\right)+W^{⊤}X^{⊤}G_{0}^{\left(t\right)}1+\beta W^{⊤}X^{⊤}L1-D^{⊤}G_{0}^{\left(t\right)}1. \end{array}$$

By setting $\frac{\partial Q_{1}}{\partial b}=0$, the closed-form solution of b can be obtained as

(23) $$b^{\left(t+1\right)}=\frac{1}{m}D^{⊤}G_{0}^{\left(t\right)}1-\frac{1}{m}W^{⊤}X^{⊤}\left(G_{0}^{\left(t\right)}+\beta L^{⊤}\right)1,$$

where $m=1^{⊤}G_{0}^{\left(t\right)}1+\beta 1^{⊤}L1$.

#### 3.3.2. Update **W**

By substituting the closed-form solution of **b** into (18), the optimization problem of W is reformulated as:

(24) $$\begin{array}{c} \min_{W}L_{2}={∥RXW-HD^{⊤}∥}_{2,1}+\alpha {∥W∥}_{2,1}+\beta \mathrm{tr}\;\left({\left(RXW-\left(H-1\right)D\right)}^{⊤}L\left(RXW-\left(H-1\right)D\right)\right), \end{array}$$

where $R=I-\frac{1}{m}\;11^{⊤}\left(G_{0}^{\left(t\right)}+\beta L\right)$ and $H=I-\frac{1}{m}\;11^{⊤}G_{0}^{\left(t\right)}$. In the same manner, the update of W is obtained via the surrogate function $Q_{2}$ associated with $L_{2}$:

(25) $$\begin{array}{cc} Q_{2} & \left(W|W^{\left(t\right)}\right)=\mathrm{tr}\;\left({\left(RXW-HD\right)}^{⊤}G_{0}^{\left(t\right)}\left(RXW-HD\right)\right)+\alpha \mathrm{tr}\left(W^{⊤}G_{1}^{\left(t\right)}W\right)\\ \; & +\beta \mathrm{tr}\;\left({\left(RXW-\left(H-1\right)D\right)}^{⊤}L\left(RXW-\left(H-1\right)D\right)\right), \end{array}$$

the definition of $G_{0}^{\left(t\right)}$ is given in (21), and that of $G_{1}^{\left(t\right)}$ is as follows:

(26) $$G_{1}^{\left(t\right)}=\frac{1}{2}\mathrm{diag}\left(∥W_{i}{∥}_{2}^{-1}\right).$$

Taking the derivative of $Q_{2}$ with respect to W and defining E=RX, we obtain

(27) $$\begin{array}{c} \frac{\partial Q_{2}}{\partial W}=\left(E^{⊤}\left(G_{0}^{\left(t\right)}+\beta L\right)E+\alpha G_{1}^{\left(t\right)}\right)W-\beta E^{⊤}L\left(H-1\right)D-E^{⊤}G_{0}^{\left(t\right)}HD. \end{array}$$

By setting the derivative in (27) to zero, the update of W admits the following closed-form solution:

(28) $$\begin{array}{cc} W^{\left(t+1\right)}= & {\left(E^{⊤}\left(G_{0}^{\left(t\right)}+\beta L\right)E+\alpha G_{1}^{\left(t\right)}\right)}^{-1}E^{⊤}\left(G_{0}^{\left(t\right)}H+\beta L\left(H-1\right)\right)D. \end{array}$$

#### 3.3.3. Update **S**

By discarding the terms in (18) that are irrelevant to **S**, the objective function can be written as:

(29) $$\begin{array}{cc} \min_{S}L_{3}= & 2\mathrm{Tr}{\left(XW+1b^{⊤}\right)}^{⊤}L\left(XW+1b^{⊤}\right)+2\gamma \sum_{i=1}^{n}\sum_{j=1}^{n}\left(S_{ij}\log S_{ij}\right)\\ \; & s.t.\sum_{j=1}^{n}S_{ij}=1,\;S_{ij}\geq 0. \end{array}$$

Based on (15), the Lagrangian corresponding to (29) can be formulated as

(30) $$\begin{array}{cc} L_{3}\left(S\right) & =\sum_{i=1}^{n}\sum_{j=1}^{n}S_{ij}{∥{\left(XW+1b^{⊤}\right)}_{i}-{\left(XW+1b^{⊤}\right)}_{j}∥}_{2}^{2}+2\gamma \sum_{i=1}^{n}\sum_{j=1}^{n}S_{ij}\log S_{ij}\\ \; & \;-\sum_{i=1}^{n}\psi_{i}\left(\sum_{j=1}^{n}S_{ij}-1\right)-\sum_{i=1}^{n}\sum_{j=1}^{n}\pi_{ij}S_{ij}, \end{array}$$

where the Lagrange multipliers are denoted by $\Psi =\{\psi_{i}|i=1,\ldots ,n\}$ and $\Pi =\{\pi_{ij}|i,j=1,\ldots ,n\}$. According to standard optimization theory, the solution must satisfy the Karush–Kuhn–Tucker (KKT) conditions. Therefore, we have

(31) $$\left\{\begin{array}{c} {∥{\left(XW+1b^{⊤}\right)}_{i}-{\left(XW+1b^{⊤}\right)}_{j}∥}_{2}^{2}+2\gamma \left(\log S_{ij}+1\right)-\psi_{i}-\pi_{ij}=0,\\ S_{ij}\geq 0,\;\pi_{ij}\geq 0,\;\pi_{ij}S_{ij}=0,\;\sum_{j=1}^{n}S_{ij}=1. \end{array}\right.$$

From these conditions, the closed-form solution for $S_{ij}$ can be directly obtained as

(32) $$S_{ij}=\frac{\exp(-\frac{∥{\left(XW+1b^{⊤}\right)}_{i}-{\left(XW+1b^{⊤}\right)}_{j}{∥}_{2}^{2}}{2\gamma })}{\sum_{j=1}^{n}\exp\left(-\frac{∥{\left(XW+1b^{⊤}\right)}_{i}-{\left(XW+1b^{⊤}\right)}_{j}{∥}_{2}^{2}}{2\gamma }\right)}.$$

Based on the above formulation and optimization strategy, the overall procedure of PML-FSMNG can be summarized as Algorithm 2.
**Algorithm 2** PML-FSMNG  1:**Input:** Dataset $X\in R^{n\times d}$, label distribution matrix $D\in R^{n\times q}$, parameter α, β, γ  2:**Output:** Top ranked features  3:Initialize t←1. W←rand(d,q), $b^{⊤}\leftarrow \mathrm{rand}\left(1,q\right)$, S←rand(n,n),  4:**while** not convergence **do**  5:    $G_{0}^{\left(t\right)}\leftarrow \frac{1}{2}\mathrm{diag}\left(∥{\left(XW+1b^{⊤}-D\right)}_{i}{∥}_{2}^{-1}\right)$  6:    update $b^{\left(t+1\right)}$ by (23)  7:    $G_{1}^{\left(t\right)}\leftarrow \frac{1}{2}\mathrm{diag}\left(∥W_{i}{∥}_{2}^{-1}\right)$  8:    $m\leftarrow 1^{⊤}G_{0}^{\left(t\right)}1+\beta 1^{⊤}L1$  9:    $R\leftarrow I-\frac{1}{m}\;11^{⊤}\left(G_{0}^{\left(t\right)}+\beta L\right)$, $H\leftarrow I-\frac{1}{m}\;11^{⊤}G_{0}^{\left(t\right)}$10:    E←RX11:    update $W^{\left(t+1\right)}$ by  (28)12:    update $S^{\left(t+1\right)}$ by  (32)13:    t←t+114:**end while**15:$W\leftarrow W^{\left(t\right)}$16:Rank features with ${∥w_{i.}∥}_{2}\left(i\in \left\{1,2,\ldots ,d\right\}\right)$

## 4. Experiments

In this section, we conduct extensive experiments on both synthetic and real-world datasets to evaluate the proposed PML-FSMNG method against seven representative approaches. To comprehensively assess the effectiveness of all compared methods, seven commonly used evaluation criteria are adopted, including Hamming Loss, Ranking Loss, One-error, Coverage, Average Precision, Micro-F1, and Subset Accuracy. The formal definitions of these metrics can be found in [38,39]. Furthermore, extensive investigations are conducted through computational complexity analysis and parameter sensitivity examination to further validate the robustness and efficiency of the proposed framework.

### 4.1. Datasets

We select four datasets (Publicly available at http://www.uco.es/kdis/mllresources/, accessed on 25 December 2025.) (i.e., Emotions, Image, VirusGo, and Yeast) and six real-world datasets (Publicly available at https://palm.seu.edu.cn/zhangml/, accessed on 25 December 2025) (i.e., Mirflickr, Music-emotion, Music-style, YeastBP, YeastCC, and YeastMF) for empirical evaluation. To assess the performance stability of the proposed method under varying noise conditions, the first four datasets are evaluated with manually injected label noise at levels of 20%, 50%, 150%, and 200%, respectively. In contrast, the latter six are standard real-world datasets that naturally exhibit partial multi-label characteristics, which are adopted to further demonstrate the effectiveness of our method in practical scenarios. Table 2 provides a detailed summary of these datasets, where “#Ins.” denotes the number of instances, “#Fea.” and “#Lab.” represent the numbers of features and labels respectively; “Label card.” indicates the average number of labels per instance, and “Label dens.” denotes the label density.

### 4.2. Experiment Settings

For multi-label prediction, MLKNN [40] is adopted as the classifier with parameters set to K=10 and S=1. The reported performance is averaged over five-fold cross-validation [41]. Furthermore, comparisons with seven representative algorithms are conducted to further validate the effectiveness of the proposed method. All algorithms are briefly introduced and tuned to search for optimal solutions within the following parameter ranges.

(1) **SCLS** [22]: A parameter-free relevance evaluation strategy that models conditional feature correlations for improved multi-label prediction.
(2) **RFSFS** [23]: Robust feature selection with flexible sparse regularization, with α,β,γ∈{0.01,0.1,0.3,0.5, 0.7,0.9,1.0}.
(3) **LCIFS** [24]: Feature selection via manifold-based regression and adaptive spectral graph learning to jointly exploit label correlations and control feature redundancy, with $\alpha ,\beta ,\gamma ,\lambda \in \{10^{-3},10^{-2},\cdots ,10^{3}\}$.
(4) **PMLFS** [25]: Partial multi-label feature selection via label correlation modeling and noisy-label sparsity discrimination with manifold regularization and $\ell_{2,1}$-norm constraint, with $\lambda_{1},\lambda_{2},\lambda_{3},\lambda_{4}\in \left\{10^{-3},10^{-2},\cdots ,10^{3}\right\}$.
(5) **PML-FSSO** [26]: Partial multi-label feature selection via joint label–feature subspace learning with linear weighted modeling and low-dimensional label matrix decomposition for noise suppression, with $\alpha ,\beta ,\gamma ,\delta \in \{10^{-3},10^{-2},\cdots ,10^{3}\}$.
(6) **PML-FSMIR** [29]: Feature selection for partial multi-label learning via noise-resistant label relationship modeling and connectivity-guided reweighted low-rank optimization, with $\alpha ,\beta ,\gamma \in \{10^{-3},10^{-2},\cdots ,10^{3}\}$.
(7) **PMSNE** [28]: Partial multi-label feature selection via robust label enhancement and sparse smoothness-constrained reconstruction with $\ell_{2,1}$-norm regularization, with $\alpha ,\beta ,\gamma ,\delta \in \{10^{-3},10^{-2},\cdots ,10^{3}\}$.
(8) **PML-FSMNG** (The code is available at https://github.com/Cao-yifan/PML_FSMNG, accessed on 29 March 2026): For PML-FSMNG, the base scale is defined as $\theta =\frac{1}{\sqrt{d}}\sum_{j=1}^{d}\sigma_{j}$, where $\sigma_{j}$ denotes the standard deviation of the *j*-th feature. Three neighborhood scales $\left\{\frac{1}{2}\theta ,\;\theta ,\;2\theta \right\}$ are used for multi-scale construction, and the regularization parameters α, β, and γ are tuned from $\left\{10^{-3},10^{-2},\cdots ,10^{3}\right\}$.

### 4.3. Results and Analysis

Table 3 and Table 4 report the optimal results of eight evaluation metrics obtained by different methods on ten datasets when selecting the top 20% of features. The best performance on each dataset is highlighted in bold and the suboptimal values are underlined. It can be observed that PML-FSMNG achieves the best performance in 82.9% of the cases. Even on the few datasets where PML-FSMNG does not attain the top result, its performance remains among the leading methods. In terms of Hamming Loss, PML-FSMNG achieves the best performance on eight datasets and attains second-best results on Emotions and YeastMF, comparable to PMSNE and PMLFS. In terms of Ranking Loss, PML-FSMNG achieves the best performance on eight datasets. In particular, it outperforms the second-best method by 1.2% on the Music-emotion dataset, and attains second-best results on Yeast and YeastCC, comparable to PMLFS and PMSNE. For One-error, PML-FSMNG ranks first on eight datasets, with improvements of 1.7% and 1.3% on Image and Music-emotion, respectively. Regarding Coverage, it achieves the best results on eight datasets, improving by 1.1% on Music-emotion and 1.0% on YeastCC, and obtains the second-best performance on YeastMF. For Average Precision, PML-FSMNG ranks first on nine datasets and second on one dataset, with gains of 1.0% on Image and 1.3% on Music-emotion. In terms of Micro-F1, it achieves the best performance on eight datasets and the second-best on two datasets, notably improving by 2.5% on Music-emotion and 1.7% on YeastCC. For Subset Accuracy, PML-FSMNG ranks first on nine datasets and second on one dataset.

To better understand these results, we further analyze the characteristics of the datasets and the underlying reasons for the observed performance patterns. Specifically, the performance of PML-FSMNG is closely related to the consistency between feature similarity and label distribution within local neighborhoods. On datasets such as Emotions, Image, and Music-emotion, which exhibit moderate label density and relatively clear local label patterns, the proposed entropy-weighted multi-scale neighborhood modeling can effectively capture feature–label consistency, leading to superior performance. However, on datasets such as YeastCC and YeastMF, the performance gain is less pronounced. These datasets are characterized by extremely low label density (around 0.025–0.027), indicating that each instance is associated with very few positive labels. In such scenarios, due to the sparsity of label assignments, the probability that neighboring samples share common labels becomes very low, even if they are close in the feature space. Consequently, the overlap of relevant labels within local neighborhoods is often insufficient to form consistent label patterns, leading to weak feature–label alignment. Consequently, heterogeneous labels within neighborhood granules elevate entropy across scales, which diminishes both the discriminability of $D^{\theta_{k}}$ and the impact of entropy-based scale weighting. Furthermore, the extremely sparse label structure limits the benefit of local neighborhood modeling, as even feature-similar neighbors often provide insufficient label information to establish reliable label guidance. This explains why the improvement of PML-FSMNG is less significant on these datasets, particularly for ranking-based metrics such as Coverage and Average Precision.

**Figure 4:**
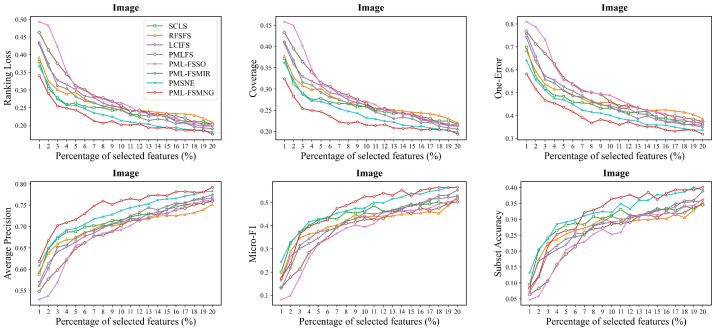
Performance comparison of PML-FSMNG against seven baseline methods on the **Image** dataset. The six subfigures illustrate the performance trends in terms of Ranking Loss, One-Error, Coverage, Average Precision, Micro-F1, and Subset Accuracy as the percentage of selected features increases from 1% to 20%.

**Figure 5:**
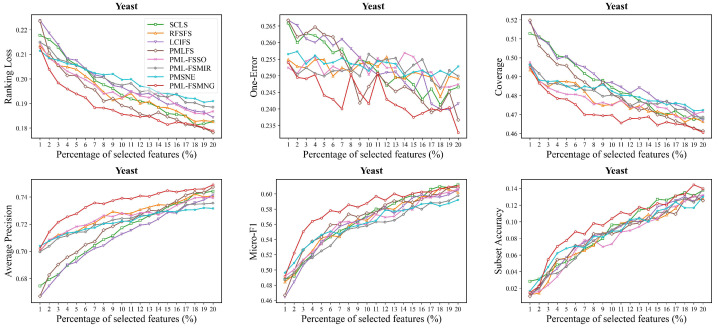
Performance comparison on the **Yeast** dataset. The baseline methods, evaluation metrics, and feature selection percentages are identical to those detailed in Figure 4.

**Figure 6:**
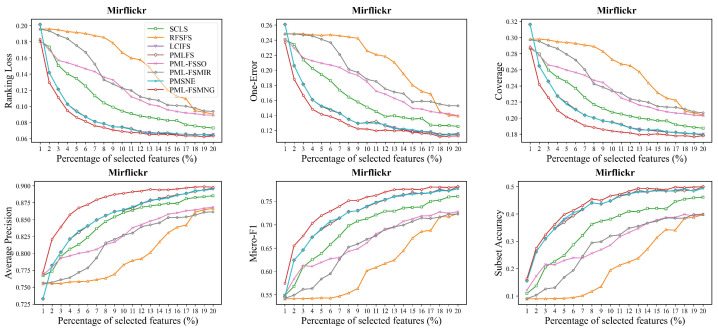
Performance comparison on the **Mirflickr** dataset (similar setup to Figure 4).

**Figure 7:**
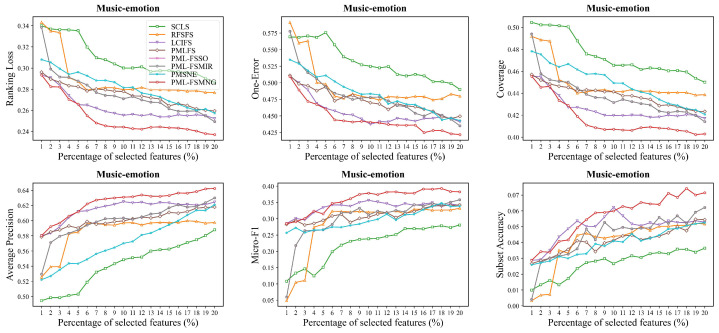
Performance comparison on the **Music-emotion** dataset (similar setup to Figure 4).

**Figure 8:**
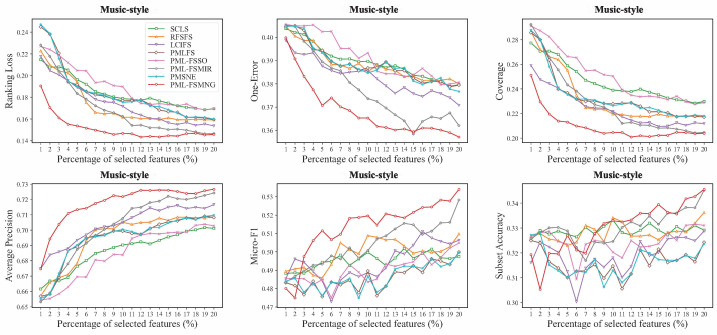
Performance comparison on the **Music-style** dataset (similar setup to Figure 4).

Moreover, we compared PML-FSMNG with seven other methods across five datasets (Image, Yeast, Mirflickr, Music-emotion and Music-style) in Figure 4, Figure 5, Figure 6, Figure 7 and Figure 8, under varying numbers of selected features ranging from 1% to 20%. The results show that, regardless of the number of selected features, PML-FSMNG consistently outperforms the competing methods. A general trend across all five datasets reveals that as the percentage of selected features increases from 1% to approximately 10%, there is a sharp improvement in performance for most methods. Beyond the 10% threshold, the performance curves tend to stabilize, indicating that the most discriminative information has been successfully captured early on. PML-FSMNG consistently dominates across all six evaluation metrics. Notably, on Mirflickr and Music-style, it establishes a significant performance gap over baselines even when selecting only 2% to 5% of features. This firmly demonstrates our method’s robustness in extracting highly compact and strongly discriminative feature subsets for partial multi-label learning.discriminative feature subset for partial multi-label learning.

**Figure 9:**
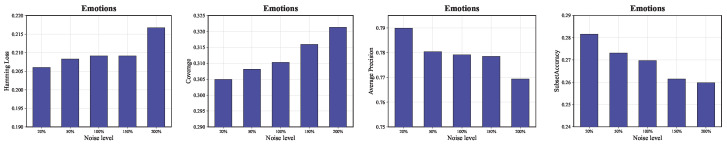
Performance of the proposed method under different noise levels on the **Emotions** dataset.

Furthermore, to investigate the robustness of PML-FSMNG under varying degrees of label corruption, we conducted an additional experiment on the Emotions dataset by manually injecting label noise ranging from 20% to 200%. The results, illustrated in Figure 9, reveal that as the noise level increases, Hamming Loss and Coverage show an upward trend, while Average Precision and Subset Accuracy decrease. Nevertheless, the graceful degradation in performance demonstrates that our method maintains robust learning capabilities and does not collapse even under extreme noise conditions (e.g., 150% and 200%). This resilience further confirms that the entropy-guided weighting mechanism effectively mitigates the impact of noisy labels during the feature selection process.

To provide a comprehensive statistical comparison among multiple competing algorithms, a two-stage hypothesis testing procedure adopted. In the first stage, we applied the Friedman test [42], a non-parametric statistical method designed for comparing several related samples based on their ranking performance. Assuming the null hypothesis that all competing methods exhibit identical performance, the corresponding Friedman statistic $F_{F}$ approximately follows an *F*-distribution with (k−1) and (k−1)(N−1) degrees of freedom. The statistic is computed as

(33) $$\begin{array}{cc} F_{F} & =\frac{\left(N-1\right)\chi_{F}^{2}}{N\left(k-1\right)-\chi_{F}^{2}}, \end{array}$$

(34) $$\begin{array}{cc} \chi_{F}^{2} & =\frac{12N}{k(k+1)}\left(\sum_{j=1}^{k}R_{j}^{2}-\frac{k{\left(k+1\right)}^{2}}{4}\right), \end{array}$$

here, *k* represents the number of methods under comparison, *N* the number of datasets, and $R_{j}$ the mean rank assigned to the *j*-th method. In our experiments, with k=8 and N=10, the critical value of $F_{F}$ is determined to be 2.1588 at a 0.05 significance level. As shown in Table 5, all Friedman statistics are considerably greater than the critical value of 2.1588. These results provide strong evidence to reject the null hypothesis, thereby confirming the existence of statistically significant performance differences among the compared methods.

**Figure 10:**
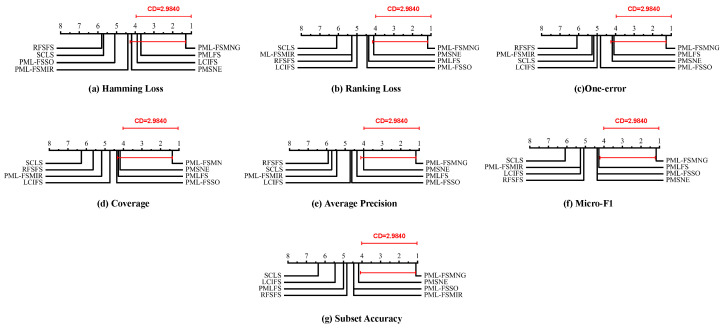
Comparison of PML-FSMNG with 7 other methods using the Bonferroni-Dunn test across all evaluation metrics, with CD = 2.9840 at a significance level of 0.05.

Based on these results, we proceeded with post-hoc analysis using the Bonferroni-Dunn test [43] to examine performance differences between PML-FSMNG and other comparative approaches. The significance of differences between PML-FSMNG and each comparison method was determined using the critical difference (CD) metric:

(35) $$CD=q_{\alpha }\sqrt{\frac{k(k+1)}{6N}},$$

where $q_{\alpha }=2.690$ at the significance level α=0.05. In our experiments, with k=8 and N=10, the critical difference (CD) value is determined to be 2.9840. As shown in Figure 10, the CD diagrams depict the comparative rankings of various methods across multiple evaluation metrics, and the CD value of 2.9840 serves as a statistical threshold, indicating that a ranking difference exceeding this threshold corresponds to a statistically significant difference in performance between the two methods.

### 4.4. Computational Complexity Analysis

Let *n*, *d*, and *q* denote the numbers of samples, features, and labels, respectively, and let *K* be the number of neighborhood scales.

Constructing the multi-scale neighborhood granules requires computing pairwise distances in the *d*-dimensional feature space, which costs $O(n^{2}d)$ per scale. Therefore, the construction of the multi-scale label distribution requires $O(Kn^{2}d)$.

For the optimization stage, updating b involves matrix–vector multiplications and requires $O(ndq+n^{2})$ time. Updating W requires forming $E^{⊤}\left(G_{0}+\beta L\right)E$, which costs $O(n^{2}d+nd^{2})$, and solving a d×d linear system with complexity $O(d^{3})$. Updating S requires computing pairwise distances in the predicted label space, resulting in a cost of $O(n^{2}q)$. Consequently, the overall time complexity of the proposed method is $O\left(Kn^{2}d+ndq+d^{3}+n^{2}q\right)$. In typical multi-label scenarios, q≪d and *K* is a small constant. Thus, the complexity can be simplified to $O(n^{2}d+d^{3})$. Since *d* is typically smaller than *n* in feature selection tasks, the dominant term becomes $O(n^{2}d)$, indicating that the method scales quadratically with respect to the number of samples and linearly with respect to the feature dimension, which is computationally manageable in practice.

To provide a clear comparison of computational efficiency, the time complexities of PML-FSMNG and several representative partial multi-label feature selection methods are summarized in Table 6, where $\tilde{d}$ denotes the number of selected features in SCLS and *k* represents the predefined low-rank dimension in PML-FSMIR.

To further evaluate the computational efficiency of the proposed method, we report the empirical running time comparisons with several representative baseline methods in Table 7. All experiments were conducted in Matlab R2020a on a PC equipped with a 13th Gen Intel(R) Core(TM) i7-13700K CPU and 32 GB of RAM. The reported results correspond to the average running time over five-fold cross-validation. As shown in Table 7, the proposed method achieves competitive runtime performance across different datasets. Specifically, compared with SCLS, our method requires less computational time on most datasets, particularly on large-scale datasets such as **Mirflickr**, **Music-emotion**, and **Music-style**, demonstrating its efficiency. Compared with more computationally intensive method LCIFS, the proposed method is significantly faster across almost all datasets, indicating its advantage in handling high-dimensional or complex data. In addition, our method is also more efficient than PML-FSSO and PMLFS on several datasets, especially as the data size increases. Although our method is slightly slower than methods such as RFSFS, PML-FSMIR and PMSNE on some datasets, the difference remains within an acceptable range. Overall, these results indicate that the proposed method achieves a favorable balance between effectiveness and computational efficiency.

### 4.5. Ablation Study and Analysis of Multi-Scale Fusion

To verify the effectiveness of the proposed multi-scale modeling and entropy-guided fusion, We conducted a series of experiments to investigate the effect of entropy-guided fusion and the impact of the number of neighborhood scales, providing a comprehensive evaluation of how these design choices influence feature selection performance.

Specifically, we construct three variants of our method on the **Music-emotion** and **Image** datasets: a version without multi-scale modeling, which uses only the base scale θ; a version without entropy-guided weighting, which preserves multiple scales but combines them using uniform weights; and the full model, which incorporates both multi-scale modeling and entropy-guided fusion.

**Figure 11:**
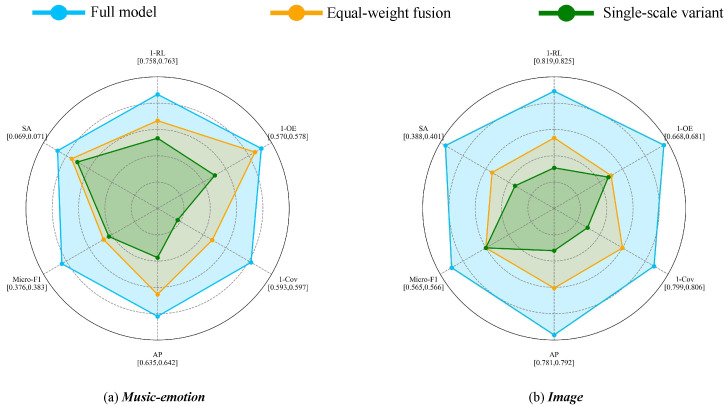
Ablation study results at 20% feature selection: performance comparison of the proposed method variants. The variants include a version without multi-scale modeling, a version without entropy-guided weighting (equal-weight fusion), and the full model combining both components.

The experimental results, illustrated in Figure 11, demonstrate the effectiveness of each component. Removing the multi-scale mechanism leads to a noticeable performance drop across the six evaluation metrics, highlighting that capturing neighborhood structures at multiple resolutions is crucial. Similarly, replacing entropy-guided fusion with equal-weight averaging also reduces performance, demonstrating that entropy-based adaptive weighting plays a critical role. The full model consistently achieves the best performance, confirming that multi-scale modeling and entropy-guided fusion complement each other and jointly contribute to improved results.

**Figure 12:**
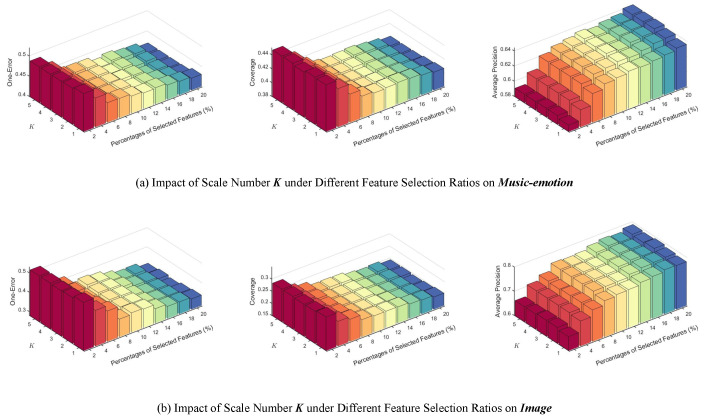
Impact of the number of neighborhood scales *K* on performance under different feature selection percentages.

In addition, we further analyzed the influence of the number of scales *K* on the **Music-emotion** and **Image** datasets. In these experiments, *K* varies from 1 to 5, and the corresponding neighborhood scales are defined as:

(36) $$\Theta_{K}=\left\{2^{\;k-\lfloor K/2\rfloor -1}\;\theta \;|\;k=1,2,\ldots ,K\right\},$$

forming a geometric progression centered at θ that covers neighborhood structures from fine to coarse resolutions. The base scale θ is adaptively determined according to the data distribution:

(37) $$\theta =\frac{1}{\sqrt{d}}\sum_{j=1}^{d}\sigma_{j},$$

where $\sigma_{j}$ denotes the standard deviation of the *j*-th feature. As illustrated in Figure 12, across different percentages of selected features, the algorithm performs the worst when only a single scale is used. The algorithm’s performance is competitive when the number of scales is 3 and 4, while further increasing *K* yields only marginal gains with higher computational cost. These observations indicate that incorporating multiple neighborhood scales is beneficial, and that a small number of scales is sufficient in practice.

### 4.6. Parameter Sensitivity Analysis

The proposed PML-FSMNG involves three key parameters: the sparsity parameter α, the graph regularization coefficient β, and the entropy regularization parameter γ. Specifically, α enforces row-wise sparsity on the feature weight matrix, β preserves geometric consistency via graph Laplacian regularization, and γ regulates the adaptivity of the similarity matrix through entropy regularization.

**Figure 13:**
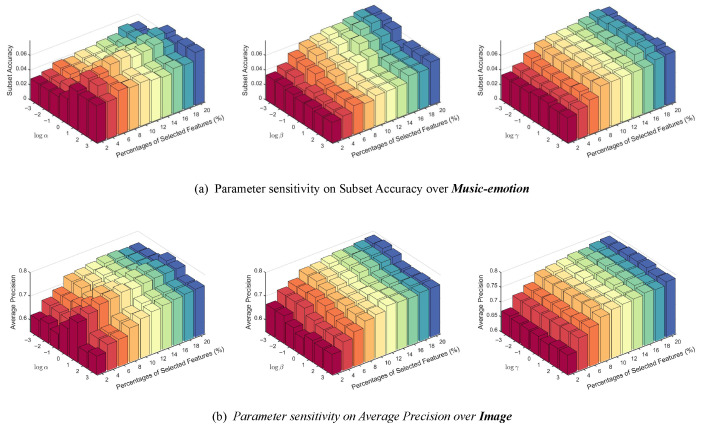
Parameter sensitivity analysis of α, β, and γ across different datasets and evaluation metrics.

Sensitivity analysis is conducted on the **Music-emotion** and **Image** datasets, where Subset Accuracy and Average Precision are adopted as evaluation metrics, respectively. For each parameter, its value is varied while the remaining parameters are fixed at their respective optimal settings. As illustrated in Figure 13, the performance remains stable across a broad range of parameter values on both datasets, demonstrating the robustness of PML-FSMNG. In particular, similar trends are consistently observed across different datasets and evaluation metrics. For α, moderate values achieve the best trade-off between sparsity and data fitting, while excessively large values tend to over-penalize feature weights and degrade performance. For β, incorporating geometric regularization helps maintain stable performance by preserving the intrinsic data structure, whereas overly large values may slightly restrict model flexibility. For γ, moderate entropy regularization yields the most reliable results, while too small or too large values weaken the adaptivity of the learned similarity structure.

These consistent observations across datasets confirm that the model exhibits strong robustness with respect to parameter variations. Consequently, we establish a heuristic-based default configuration of α=1, $\beta =10^{-3}$, and γ=1. This setting serves as a strong, out-of-the-box baseline that yields highly competitive performance without the need for exhaustive manual tuning, thereby significantly enhancing the user-friendliness of the proposed model.

## 5. Conclusions

In this paper, we present PML-FSMNG, a unified framework for partial multi-label feature selection that jointly leverages multi-scale neighborhood modeling, entropy-driven label distribution fusion, sparse regression, and adaptive graph learning. By capturing instance-level structures across multiple neighborhood scales and emphasizing label-consistent scales via entropy weighting, the proposed method provides a robust soft supervisory representation for handling label ambiguity. The integration of $\ell_{2,1}$-norm sparsity and entropy-regularized graph learning further enhances robustness and geometric consistency during optimization. Experimental results confirm the superiority of the proposed approach over existing methods, highlighting the importance of multi-granularity structural modeling in partial multi-label learning. These findings suggest that incorporating multi-scale structural information and adaptive information-theoretic mechanisms offers a promising direction for robust feature selection under uncertain supervision.

## Figures and Tables

**Table 1:** Comparison of neighborhood entropy and assigned weight for each scale under clean and noisy conditions, with bold values indicating the best performance for each scale.

Condition	$H^{\theta_{1}}$	$H^{\theta_{2}}$	$H^{\theta_{3}}$	$w^{\theta_{1}}$	$w^{\theta_{2}}$	$w^{\theta_{3}}$
Noise-free	0	0.523	0.799	**0.490**	0.290	0.220
Noise-injected	0.918	0.787	0.945	0.321	**0.366**	0.313

**Table 2:** Details of datasets.

Source	Dataset	Noise Level	#Ins.	#Fea.	#Lab.	Label Card.	Label Dens.	Domain
Synthetic	Emotions	20%	593	72	6	1.8685	0.3114	Music
	Image	50%	2000	294	5	1.2360	0.2472	Images
	VirusGo	150%	207	749	6	1.2174	0.2029	Biology
	Yeast	200%	2417	103	14	4.2371	0.3026	Biology
Real-world	Mirflickr	–	10,433	100	7	1.7744	0.2535	Image
	Music-emotion	–	6833	98	11	2.4235	0.2203	Music
	Music-style	–	6839	98	10	1.4404	0.1440	Music
	YeastBP	–	6139	6139	217	5.5367	0.0255	Biology
	YeastCC	–	6139	6139	50	1.3476	0.0270	Biology
	YeastMF	–	6139	6139	39	1.0050	0.0258	Biology

**Table 3:** Performance comparison results of eight feature selection methods on Hamming Loss, Ranking Loss, One-Error and Coverage (mean ± std).

Data	SCLS	RFSFS	LCIFS	PMLFS	PML-FSSO	PML-FSMIR	PMSNE	PML-FSMNG
**Hamming Loss ↓**								
Emotions	0.2105 ± 0.0126	0.2176 ± 0.0155	0.2131 ± 0.0088	0.2125 ± 0.0184	0.2097 ± 0.0082	0.2150 ± 0.0046	**0.2010 ± 0.0108**	0.2060 ± 0.0119
Image	0.1850 ± 0.0033	0.1894 ± 0.0058	0.1826 ± 0.0119	0.1902 ± 0.0111	0.1907 ± 0.0056	0.1824 ± 0.0063	0.1741 ± 0.0065	**0.1736 ± 0.0054**
VirusGo	0.0700 ± 0.0172	0.0691 ± 0.0234	0.0675 ± 0.0240	**0.0635 ± 0.0164**	0.0676 ± 0.0120	0.0675 ± 0.0188	0.0707 ± 0.0211	**0.0635 ± 0.0209**
Yeast	0.2063 ± 0.0037	0.2106 ± 0.0031	0.2071 ± 0.0021	0.2097 ± 0.0017	0.2088 ± 0.0040	0.2115 ± 0.0015	0.2120 ± 0.0036	**0.2055 ± 0.0043**
Mirflickr	0.1142 ± 0.0039	0.1294 ± 0.0032	0.1075 ± 0.0021	0.1064 ± 0.0022	0.1259 ± 0.0039	0.1278 ± 0.0064	0.1077 ± 0.0019	**0.1059 ± 0.0031**
Music-emotion	0.2055 ± 0.0065	0.2035 ± 0.0010	0.1990 ± 0.0020	0.1994 ± 0.0020	0.2012 ± 0.0018	0.1989 ± 0.0026	0.2001 ± 0.0016	**0.1950 ± 0.0025**
Music-style	0.1201 ± 0.0030	0.1205 ± 0.0016	0.1175 ± 0.0016	0.1186 ± 0.0018	0.1199 ± 0.0018	0.1161 ± 0.0016	0.1179 ± 0.0021	**0.1139 ± 0.0019**
YeastBP	0.0366 ± 0.0008	**0.0354 ± 0.0007**	0.0364 ± 0.0008	0.0356 ± 0.0010	**0.0354 ± 0.0008**	0.0362 ± 0.0009	0.0359 ± 0.0008	**0.0354 ± 0.0007**
YeastCC	0.0669 ± 0.0008	0.0666 ± 0.0010	0.0665 ± 0.0007	0.0663 ± 0.0012	0.0665 ± 0.0011	0.0665 ± 0.0006	0.0662 ± 0.0014	**0.0658 ± 0.0010**
YeastMF	0.0609 ± 0.0009	**0.0608 ± 0.0006**	0.0609 ± 0.0009	**0.0608 ± 0.0010**	0.0610 ± 0.0012	0.0609 ± 0.0010	0.0609 ± 0.0011	0.0609 ± 0.0011
**Ranking Loss ↓**								
Emotions	0.1863 ± 0.0151	0.1831 ± 0.0258	0.1766 ± 0.0098	0.1841 ± 0.0177	0.1768 ± 0.0167	0.1889 ± 0.0117	0.1733 ± 0.0128	**0.1725 ± 0.0148**
Image	0.2027 ± 0.0150	0.2078 ± 0.0133	0.1974 ± 0.0193	0.2027 ± 0.0118	0.1984 ± 0.0079	0.1901 ± 0.0162	0.1810 ± 0.0149	**0.1752 ± 0.0129**
VirusGo	0.0458 ± 0.0249	0.0516 ± 0.0215	0.0494 ± 0.0218	0.0516 ± 0.0177	0.0487 ± 0.0199	0.0490 ± 0.0224	0.0557 ± 0.0186	**0.0438 ± 0.0196**
Yeast	0.1826 ± 0.0070	0.1826 ± 0.0044	0.1844 ± 0.0097	**0.1782 ± 0.0026**	0.1868 ± 0.0074	0.1885 ± 0.0058	0.1910 ± 0.0046	0.1789 ± 0.0088
Mirflickr	0.0732 ± 0.0029	0.0903 ± 0.0013	0.0653 ± 0.0017	0.0638 ± 0.0015	0.0889 ± 0.0062	0.0937 ± 0.0058	0.0647 ± 0.0026	**0.0633 ± 0.0021**
Music-emotion	0.2862 ± 0.0267	0.2767 ± 0.0065	0.2521 ± 0.0071	0.2594 ± 0.0094	0.2670 ± 0.0107	0.2492 ± 0.0053	0.2572 ± 0.0071	**0.2370 ± 0.0088**
Music-style	0.1694 ± 0.0105	0.1592 ± 0.0041	0.1536 ± 0.0079	0.1595 ± 0.0031	0.1692 ± 0.0055	0.1464 ± 0.0034	0.1599 ± 0.0033	**0.1457 ± 0.0059**
YeastBP	0.3533 ± 0.0081	0.3133 ± 0.0085	0.3529 ± 0.0103	0.3141 ± 0.0101	0.3046 ± 0.0082	0.3486 ± 0.0075	0.3203 ± 0.0094	**0.3034 ± 0.0078**
YeastCC	0.3458 ± 0.0091	0.3442 ± 0.0148	0.3446 ± 0.0109	0.3371 ± 0.0190	0.3406 ± 0.0084	0.3448 ± 0.0035	**0.3332 ± 0.0085**	0.3365 ± 0.0160
YeastMF	0.3549 ± 0.0158	0.3548 ± 0.0285	0.3635 ± 0.0052	0.3466 ± 0.0132	0.3527 ± 0.0065	0.3547 ± 0.0068	0.3482 ± 0.0126	**0.3447 ± 0.0055**
**One-error ↓**								
Emotions	0.3001 ± 0.0376	0.3255 ± 0.0491	0.3002 ± 0.0280	0.3104 ± 0.0320	0.2902 ± 0.0406	0.3070 ± 0.0175	0.2850 ± 0.0164	**0.2834 ± 0.0332**
Image	0.3625 ± 0.0196	0.3860 ± 0.0108	0.3560 ± 0.0352	0.3740 ± 0.0161	0.3735 ± 0.0127	0.3485 ± 0.0218	0.3365 ± 0.0290	**0.3190 ± 0.0335**
VirusGo	**0.1206 ± 0.0282**	0.1397 ± 0.0479	0.1256 ± 0.0467	0.1398 ± 0.0300	0.1254 ± 0.0351	0.1302 ± 0.0259	0.1495 ± 0.0296	0.1254 ± 0.0351
Yeast	0.2466 ± 0.0172	0.2491 ± 0.0213	0.2416 ± 0.0172	0.2367 ± 0.0131	0.2474 ± 0.0183	0.2499 ± 0.0108	0.2528 ± 0.0112	**0.2329 ± 0.0197**
Mirflickr	0.1253 ± 0.0054	0.1390 ± 0.0061	0.1160 ± 0.0056	0.1143 ± 0.0037	0.1394 ± 0.0075	0.1527 ± 0.0124	0.1149 ± 0.0070	**0.1126 ± 0.0035**
Music-emotion	0.4898 ± 0.0522	0.4797 ± 0.0083	0.4410 ± 0.0108	0.4493 ± 0.0132	0.4629 ± 0.0212	0.4349 ± 0.0102	0.4429 ± 0.0071	**0.4218 ± 0.0122**
Music-style	0.3796 ± 0.0084	0.3800 ± 0.0109	0.3708 ± 0.0132	0.3794 ± 0.0134	0.3806 ± 0.0137	0.3620 ± 0.0069	0.3767 ± 0.0136	**0.3571 ± 0.0101**
YeastBP	0.8438 ± 0.0064	0.7736 ± 0.0074	0.8424 ± 0.0132	0.7764 ± 0.0091	**0.7597 ± 0.0137**	0.8255 ± 0.0088	0.7881 ± 0.0075	0.7635 ± 0.0187
YeastCC	0.7580 ± 0.0155	0.7475 ± 0.0198	0.7531 ± 0.0076	0.7202 ± 0.0293	0.7473 ± 0.0126	0.7523 ± 0.0145	0.7366 ± 0.0228	**0.7198 ± 0.0313**
YeastMF	0.8373 ± 0.0095	0.8443 ± 0.0322	0.8489 ± 0.0124	0.8204 ± 0.0096	0.8570 ± 0.0211	0.8458 ± 0.0053	0.8192 ± 0.0034	**0.8180 ± 0.0123**
**Coverage ↓**								
Emotions	0.3201 ± 0.0139	0.3128 ± 0.0246	0.3072 ± 0.0052	0.3134 ± 0.0197	0.3100 ± 0.0165	0.3207 ± 0.0132	0.3083 ± 0.0103	**0.3049 ± 0.0130**
Image	0.2168 ± 0.0142	0.2200 ± 0.0132	0.2119 ± 0.0165	0.2148 ± 0.0096	0.2126 ± 0.0073	0.2049 ± 0.0126	0.1982 ± 0.0123	**0.1936 ± 0.0099**
VirusGo	0.0803 ± 0.0288	0.0827 ± 0.0227	0.0812 ± 0.0201	0.0820 ± 0.0199	0.0811 ± 0.0230	0.0819 ± 0.0250	0.0860 ± 0.0215	**0.0747 ± 0.0211**
Yeast	0.4679 ± 0.0099	0.4662 ± 0.0069	0.4671 ± 0.0061	0.4613 ± 0.0062	0.4715 ± 0.0099	0.4686 ± 0.0069	0.4723 ± 0.0036	**0.4605 ± 0.0073**
Mirflickr	0.1874 ± 0.0020	0.2039 ± 0.0029	0.1800 ± 0.0026	0.1786 ± 0.0031	0.2030 ± 0.0053	0.2065 ± 0.0064	0.1796 ± 0.0029	**0.1779 ± 0.0037**
Music-emotion	0.4499 ± 0.0214	0.4386 ± 0.0065	0.4170 ± 0.0091	0.4233 ± 0.0079	0.4322 ± 0.0079	0.4140 ± 0.0061	0.4206 ± 0.0074	**0.4029 ± 0.0077**
Music-style	0.2298 ± 0.0110	0.2175 ± 0.0058	0.2118 ± 0.0088	0.2171 ± 0.0046	0.2289 ± 0.0072	0.2046 ± 0.0058	0.2181 ± 0.0046	**0.2039 ± 0.0060**
YeastBP	0.6290 ± 0.0074	0.5649 ± 0.0080	0.6260 ± 0.0180	0.5696 ± 0.0126	0.5536 ± 0.0204	0.6218 ± 0.0135	0.5769 ± 0.0205	**0.5525 ± 0.0101**
YeastCC	0.4936 ± 0.0092	0.4924 ± 0.0184	0.4870 ± 0.0106	0.4793 ± 0.0235	0.4787 ± 0.0129	0.4845 ± 0.0072	**0.4689 ± 0.0103**	0.4802 ± 0.0193
YeastMF	0.4578 ± 0.0180	0.4585 ± 0.0328	0.4744 ± 0.0121	**0.4487 ± 0.0169**	0.4572 ± 0.0105	0.4597 ± 0.0134	0.4534 ± 0.0112	0.4490 ± 0.0044

**Table 4:** Performance comparison results of eight feature selection methods on Average Precision, Micro-F1 and Subset Accuracy (mean ± std).

Data	SCLS	RFSFS	LCIFS	PMLFS	PML-FSSO	PML-FSMIR	PMSNE	PML-FSMNG
**Average Precision ↑**								
Emotions	0.7747 ± 0.0182	0.7726 ± 0.0269	0.7829 ± 0.0094	0.7774 ± 0.0154	0.7871 ± 0.0184	0.7766 ± 0.0109	0.7888 ± 0.0107	**0.7899 ± 0.0143**
Image	0.7621 ± 0.0160	0.7515 ± 0.0121	0.7671 ± 0.0226	0.7594 ± 0.0115	0.7605 ± 0.0077	0.7743 ± 0.0157	0.7828 ± 0.0178	**0.7923 ± 0.0176**
VirusGo	0.9213 ± 0.0290	0.9149 ± 0.0273	0.9208 ± 0.0281	0.9153 ± 0.0189	0.9225 ± 0.0200	0.9192 ± 0.0231	0.9084 ± 0.0220	**0.9261 ± 0.0243**
Yeast	0.7443 ± 0.0116	0.7409 ± 0.0104	0.7415 ± 0.0104	0.7473 ± 0.0050	0.7393 ± 0.0116	0.7355 ± 0.0053	0.7316 ± 0.0053	**0.7490 ± 0.0137**
Mirflickr	0.8853 ± 0.0037	0.8665 ± 0.0032	0.8948 ± 0.0031	0.8966 ± 0.0025	0.8683 ± 0.0066	0.8615 ± 0.0086	0.8955 ± 0.0041	**0.8978 ± 0.0030**
Music-emotion	0.5878 ± 0.0323	0.5975 ± 0.0079	0.6244 ± 0.0087	0.6178 ± 0.0122	0.6083 ± 0.0138	0.6298 ± 0.0056	0.6210 ± 0.0094	**0.6423 ± 0.0101**
Music-style	0.7011 ± 0.0088	0.7084 ± 0.0067	0.7166 ± 0.0113	0.7083 ± 0.0066	0.7028 ± 0.0077	0.7242 ± 0.0020	0.7097 ± 0.0067	**0.7266 ± 0.0079**
YeastBP	0.1665 ± 0.0075	0.2260 ± 0.0045	0.1670 ± 0.0129	0.2237 ± 0.0047	0.2401 ± 0.0076	0.1771 ± 0.0079	0.2178 ± 0.0128	**0.2425 ± 0.0070**
YeastCC	0.3080 ± 0.0110	0.3229 ± 0.0179	0.3123 ± 0.0091	0.3416 ± 0.0265	0.3230 ± 0.0134	0.3110 ± 0.0018	0.3276 ± 0.0127	**0.3435 ± 0.0241**
YeastMF	0.2742 ± 0.0109	0.2688 ± 0.0324	0.2682 ± 0.0090	**0.2913 ± 0.0146**	0.2661 ± 0.0180	0.2652 ± 0.0056	0.2881 ± 0.0120	0.2880 ± 0.0097
**Micro-F1 ↑**								
Emotions	0.6227 ± 0.0291	0.6243 ± 0.0230	0.6214 ± 0.0096	0.6288 ± 0.0335	0.6320 ± 0.0172	0.6164 ± 0.0107	**0.6559 ± 0.0188**	0.6421 ± 0.0242
Image	0.5206 ± 0.0169	0.5184 ± 0.0233	0.5281 ± 0.0450	0.5028 ± 0.0378	0.5141 ± 0.0211	0.5532 ± 0.0190	0.5652 ± 0.0153	**0.5658 ± 0.0217**
VirusGo	0.8168 ± 0.0411	0.8133 ± 0.0637	0.8223 ± 0.0608	0.8288 ± 0.0453	0.8214 ± 0.0303	0.8210 ± 0.0473	0.8128 ± 0.0574	**0.8328 ± 0.0566**
Yeast	**0.6119 ± 0.0070**	0.6020 ± 0.0029	0.6053 ± 0.0088	0.6080 ± 0.0059	0.6062 ± 0.0166	0.5976 ± 0.0090	0.5920 ± 0.0096	0.6104 ± 0.0096
Mirflickr	0.7611 ± 0.0060	0.7244 ± 0.0099	0.7774 ± 0.0037	0.7812 ± 0.0055	0.7280 ± 0.0169	0.7229 ± 0.0190	0.7770 ± 0.0032	**0.7815 ± 0.0076**
Music-emotion	0.2807 ± 0.0688	0.3319 ± 0.0085	0.3412 ± 0.0137	0.3417 ± 0.0099	0.3412 ± 0.0104	0.3580 ± 0.0241	0.3385 ± 0.0101	**0.3826 ± 0.0230**
Music-style	0.4974 ± 0.0125	0.5097 ± 0.0074	0.5063 ± 0.0063	0.4999 ± 0.0125	0.5048 ± 0.0076	0.5282 ± 0.0099	0.4995 ± 0.0119	**0.5340 ± 0.0094**
YeastBP	0.0288 ± 0.0069	0.1117 ± 0.0124	0.0428 ± 0.0109	0.1082 ± 0.0172	0.1236 ± 0.0129	0.0507 ± 0.0116	0.0854 ± 0.0071	**0.1293 ± 0.0121**
YeastCC	0.0635 ± 0.0122	0.0915 ± 0.0162	0.0720 ± 0.0149	0.0943 ± 0.0195	0.0903 ± 0.0120	0.0788 ± 0.0180	0.1034 ± 0.0303	**0.1206 ± 0.0098**
YeastMF	0.0080 ± 0.0054	0.0202 ± 0.0231	0.0093 ± 0.0044	0.0188 ± 0.0090	0.0150 ± 0.0068	0.0156 ± 0.0091	0.0324 ± 0.0045	**0.0344 ± 0.0145**
**Subset Accuracy ↑**								
Emotions	0.2597 ± 0.0430	0.2698 ± 0.0332	0.2614 ± 0.0331	0.2682 ± 0.0366	0.2749 ± 0.0292	0.2547 ± 0.0190	**0.3002 ± 0.0483**	0.2816 ± 0.0178
Image	0.3490 ± 0.0154	0.3535 ± 0.0325	0.3585 ± 0.0468	0.3450 ± 0.0354	0.3625 ± 0.0333	0.3880 ± 0.0167	0.3920 ± 0.0102	**0.4010 ± 0.0216**
VirusGo	0.6476 ± 0.0866	0.6866 ± 0.1190	0.6911 ± 0.0983	0.7059 ± 0.0791	0.6862 ± 0.0545	0.6961 ± 0.0747	0.6865 ± 0.0980	**0.7060 ± 0.1078**
Yeast	0.1378 ± 0.0098	0.1278 ± 0.0111	0.1312 ± 0.0083	0.1254 ± 0.0104	0.1316 ± 0.0180	0.1390 ± 0.0159	0.1299 ± 0.0103	**0.1398 ± 0.0125**
Mirflickr	0.4615 ± 0.0114	0.3978 ± 0.0155	0.4944 ± 0.0079	0.4995 ± 0.0109	0.3999 ± 0.0302	0.3994 ± 0.0299	0.4937 ± 0.0077	**0.4998 ± 0.0121**
Music-emotion	0.0364 ± 0.0175	0.0517 ± 0.0068	0.0528 ± 0.0058	0.0544 ± 0.0063	0.0477 ± 0.0060	0.0621 ± 0.0089	0.0525 ± 0.0078	**0.0714 ± 0.0074**
Music-style	0.3291 ± 0.0070	0.3362 ± 0.0067	0.3287 ± 0.0048	0.3242 ± 0.0093	0.3310 ± 0.0100	0.3451 ± 0.0046	0.3236 ± 0.0087	**0.3455 ± 0.0075**
YeastBP	0.0028 ± 0.0018	0.0162 ± 0.0030	0.0063 ± 0.0028	0.0132 ± 0.0040	0.0157 ± 0.0027	0.0087 ± 0.0037	0.0106 ± 0.0042	**0.0164 ± 0.0032**
YeastCC	0.0173 ± 0.0061	0.0315 ± 0.0098	0.0234 ± 0.0018	0.0259 ± 0.0045	0.0263 ± 0.0055	0.0283 ± 0.0045	0.0283 ± 0.0039	**0.0353 ± 0.0040**
YeastMF	0.0031 ± 0.0011	0.0042 ± 0.0046	0.0027 ± 0.0017	0.0100 ± 0.0089	0.0042 ± 0.0032	0.0039 ± 0.0030	0.0096 ± 0.0047	**0.0116 ± 0.0061**

**Table 5:** Friedman test statistics and critical value across evaluation metrics.

Evaluation Metric	$F_{F}$	Critical Value
Hamming Loss	4.4833	2.1588
Ranking Loss	5.5706	
One-Error	4.8221	
Coverage	5.2319	
Average Precision	5.8119	
Micro-F1	5.4296	
Subset Accuracy	6.2796	

**Table 6:** Computational complexity of different methods.

Method	Time Complexity
SCLS	$O\left(dq+d\tilde{d}\right)$
RFSFS	$O(nd^{2}+d^{2}q+ndq)$
LCIFS	$O\left(n^{3}+n^{2}d+d^{2}n+d^{3}\right)$
PMLFS	$O\left(n^{2}d+nd^{2}+nq^{2}+ndq+d^{2}q\right)$
PML-FSSO	$O\left(n^{2}d+ndq\right)$
PML-FSMIR	$O\left(knd+nq^{2}+dq^{2}\right)$
PMSNE	$O\left(n^{2}d+n^{2}q+ndq+d^{2}q+nq^{2}\right)$
PML-FSMNG	$O\left(Kn^{2}d+ndq+d^{3}+n^{2}q\right)$

**Table 7:** Running time (in seconds) of eight feature selection methods.

Data	SCLS	RFSFS	LCIFS	PMLFS	PML-FSSO	PML-FSMIR	PMSNE	OURS
Emotions	0.0151	0.0074	0.6650	0.0178	0.0332	0.0053	0.1837	0.2308
Image	0.3872	0.0167	10.5196	0.1422	0.7609	0.1198	2.4799	3.5967
VirusGo	0.5450	0.0306	0.5161	0.5580	0.4713	0.0970	0.0974	0.4122
Yeast	0.0682	0.0054	6.5574	0.0222	0.4263	0.0647	2.7619	1.7849
Mirflickr	1939.6805	0.0129	57.8930	0.0043	6.0794	0.2333	43.7303	97.0789
Music-emotion	903.6815	0.0130	51.6852	0.0064	2.1575	0.1624	17.1961	22.8995
Music-style	967.7816	0.0137	43.1117	0.0043	2.1874	0.1411	20.4853	29.2008
YeastBP	523.6046	13.9543	683.4296	532.5251	265.3128	21.4852	344.3095	309.5736
YeastCC	247.3629	7.4343	282.7126	605.7120	230.4827	11.2909	54.1246	59.9554
YeastMF	266.9288	5.8488	250.1467	583.2440	201.6546	10.7406	49.7361	59.4915

## Data Availability

Publicly available datasets were analyzed in this study. The datasets can be accessed at the following repositories: http://www.uco.es/kdis/mllresources/ (accessed on 25 December 2025) and https://palm.seu.edu.cn/zhangml/ (accessed on 25 December 2025).
